# Dermatomyositis post vaccine against SARS-COV2

**DOI:** 10.1186/s41927-022-00250-6

**Published:** 2022-04-01

**Authors:** Adolfo Camargo Coronel, Francisco Javier Jiménez Balderas, Horacio Quiñones Moya, Mario Raúl Hernández Zavala, Pedro Mandinabeitia Rodríguez, José Ramiro Hernández Vázquez, Sandy Zamora Zarco, Sergio De Jesús Aguilar Castillo

**Affiliations:** Present Address: Rheumatology Department, “Centro Medico Nacional Siglo XXI”, Mexico City, Mexico

**Keywords:** Dermatomyositis, COVID-19, Vaccine

## Abstract

**Background:**

Dermatomyositis belongs to an infrequent group of diseases predominantly found in patients older than 40 years old and is characterized by dermal and muscular findings. This disease presents itself as proximal, ascending and symmetric weakness and typical dermatosis with findings such as elevated muscle enzymes, altered electromyography and typical changes in muscle biopsy; as of today, the etiology of the disease in unknown. The COVID-19 vaccine has been a fundamental tactic to achieve control of the coronavirus (SARS CoV2), and it’s clear that the benefits of getting the vaccine overweight the risks that might come along with it. Although rare, all adverse effects should be reported, this could help us to understand the elusive pathophysiology of inflammatory idiopathic myopathy.

**Case presentation:**

In this text we will describe the case of a patient with dermatomyositis who was vaccinated against SARS CoV2 with BNT162b2 mRNA (Pfizer-BioNTech), showing a temporal relation between the vaccination and the beginning of her symptoms. We realized all the diagnostic approach to the suspected disease including electromyography, muscle biopsy and laboratory findings, corroborating the diagnosis. The patient received standard treatment for this disease (steroid therapy) and have a classic slow improvement.

**Conclusions:**

Although it´s not possible to confirm a direct correlation between the vaccine and the onset of the disease, we considered that there are enough data to suspect that this could be a trigger event and therefore should always be considered a possible cause for a case of inflammatory idiopathic myopathy.

## Background

Idiopathic Inflammatory myopathies (IIM; also known collectively as myopathies) are a heterogeneous group of immunomediated pathologies that present chronic muscular inflammation that leads up to muscle weakness [1]. They are subdivided in 4 groups: dermatomyositis (DM), polymyositis (PM), necrotizing autoimmune myopathy (NAM) and inclusion bodies myopathy (IBM). These subtypes are based primarily on the most predominant affection on either the skin or muscle [1, 2].

The pathogenesis is complicated and currently is not fully known but it responds well to immunosuppressant therapy which means there is an autoimmunne response at the core of the disease.

Vaccines are a major contribution to public health on modern age and act as a powerful stimulus to the immune system which, theoretically, has the potential to induce or exacerbate immune processes shown as serologic findings or clinical autoimmune diseases.

Even though a clear relation between the inflammatory myopathies and vaccination hasn’t been found, there are many case reports of vaccine associated myopathies. Any described association between immunization and autoimmune reaction is mostly temporal, not causal. Several trials and studies trying to prove any causal association between the two has had negative results. These findings are backed up by the fact that there has been no increased incidence of autoimmune diseases in the period of time surrounding big vaccination campaigns [3].

This studies, however, lack the statistical power to rule out any “extremely rare” causal association suggested by the case reports. It’s not unreasonable to propose that vaccines could induce several immune reactions in genetically predisposed individuals that could add up to an autoimmune disease, still, there is no irrefutable evidence that shows a causal association between vaccination and autoimmunity.

Up until august 23 of 2021, the VAERS (Vaccine Adverse Event Reporting System) had registered 77 cases of vaccine associated dermatomyositis, of which 17 of them (22.7%) were attributed to SARS-CoV2 vaccines (7 from Pfizer-BioNtech, 9 from Moderna and 1 from Jansen); 3 cases of inclusion bodies myopathy of which 2 (66.7%) were associated to SARS-CoV2 vaccines (1 from Moderna, 1 from Pfizer-BioNTech); 26 cases of polymyositis of which 6 (23.06%) were associated to SARS-CoV2vaccines (4 from Moderna and 2 from Pfizer- BioNTech); and 4 cases of immunomediated myositis, all of which were associated to the SARS-CoV2 vaccine (3 from Moderna and 1 from Pfizer-BioNTech) [4]. It’s worth mentioning that these reports are limited to USA and that any report just means a temporal association and not a causal one.

As of today, COVID19 vaccination has been related to many adverse effects (anaphylactic reactions, Guillain- Barre syndrome, pulmonary thromboembolism, etc.) [5–11], but to our knowledge, there has not been many COVID19 vaccine associated myopathies case reports aside from the VAERS reports, and we believe it’s important to know the clinical course of these possible complications of the vaccine. It’s important to clarify however, that up to this point, more than 60% of north Americans (approximately 192 million people) [12] has received at least one dose of the COVID19 vaccine and there has only been 32 myopathy cases reported in the VAERS (0.00016% of the vaccinated population), so, in case of proving causal relation the risk of presenting this complication would be very low and wouldn’t compare to the potential benefits of the vaccine [13].

## Case presentation

76 year old woman without a family or personal history of autoimmune disease and the diagnosis of systemic arterial hypertension, grade II obesity (BMI: 39.9) and recent SARS CoV2 infection (02/06/2021) that didn’t require oxygen therapy nor hospitalization. She currently uses nifedipine and metoprolol as her antihypertensive treatment and denies any current use of steroids, statins or recent changes in her medication. She gets the first dose of BNT162b2 mRNA vaccine in March (03/07/2021) and the second dose in April (04/27/2021).

One day after the second dose of the vaccine she presents disseminated dermatosis with erythematous plaques in shoulders, cleavage, abdomen, hips and tights along with bilateral muscle aches 10/10 in VAS in both pelvic and scapular members. 7 weeks later she noticed edema and a violaceous coloration in both eyelids accompanied by weakness with a symmetrical pattern affecting the proximal muscles of the four extremities that limited her daily life activities and later on started with dysphagia to both solids and liquids that made her seek medical assistance in our hospital.

**Fig. 1:**
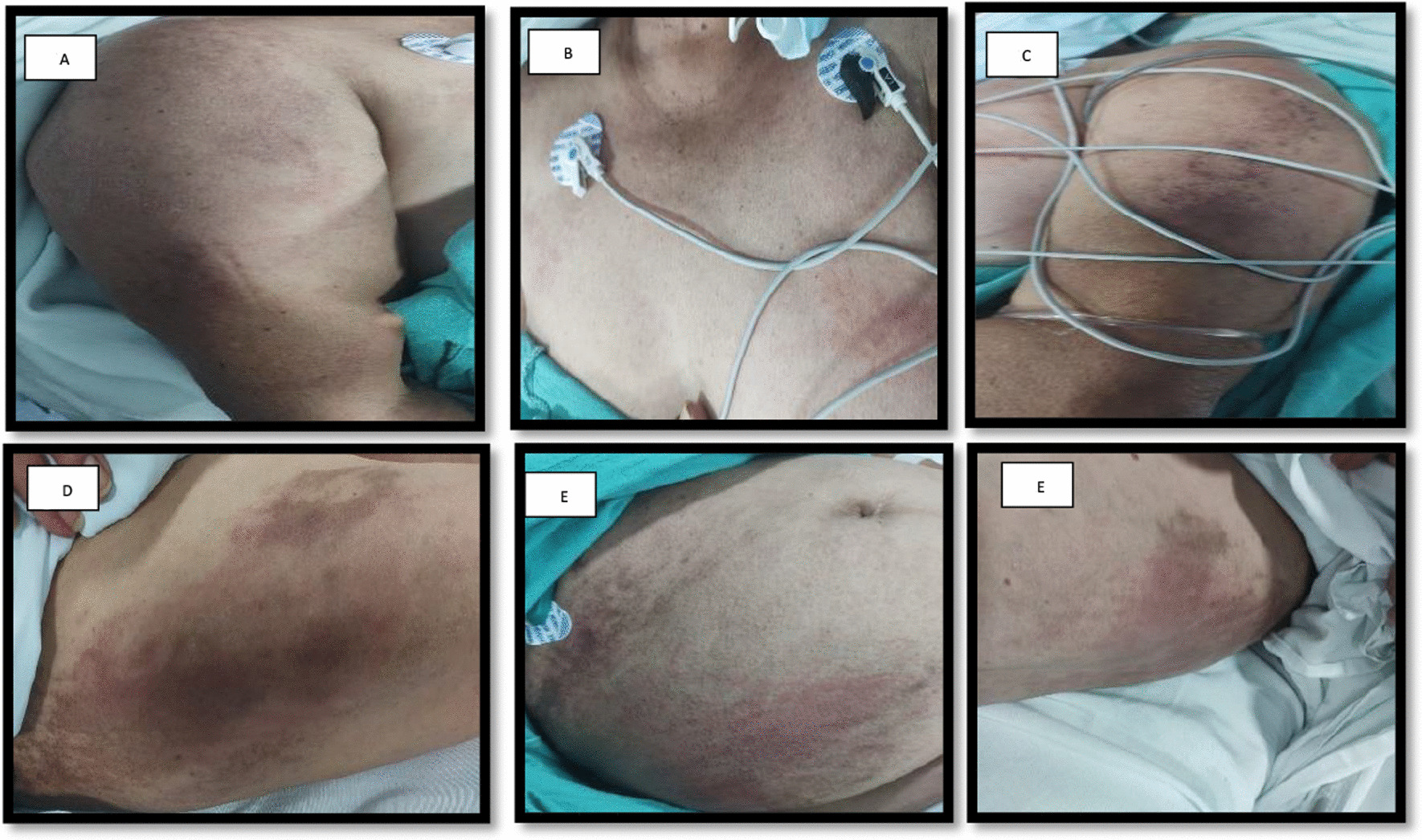
Disseminated dermatosis with erytematovolaceous plaques with hyperpigmentation areas in proximal bilateral region of both arms and tights. See the Holster Sign in both tights and the “V” sign on the cleavage with hyperpigmentation areas. **a** Right arm; **b** anterior thorax; **c** left arm; **d** right tight; **e** abdomen; **f** left tight

She arrived at the emergency department with the next vitals: Blood pressure: 111/74 mmHg, heart rate: 80 bpm, respiratory rate: 18 breaths per minute, body temperature: 97.16°F, oxygen saturation: 94%. The skin had non-confluent, well defined erytematoviolaceous plaques with hyperpigmentation and scratch marks in the proximal third of arms and tights (Fig. 1). Strength was tested with MMT8 score with 58/75 and there were no changes on osteotendinous reflexes or sensitivity.

Initial laboratory findings evidenced elevation of muscles enzymes, transaminases and lactic dehydrogenase.

The patient got 8.4 points of the EULAR-ACR 2017 criteria [14]: Age of onset ≥40 years; Objective symmetric weakness, usually progressive, of the proximal upper extremities; Objective symmetric weakness, usually progressive, of the proximal lower extremities; Neck flexors are relatively weaker then extensors; In lower extremities proximal muscles are relatively weaker than distal ones; Dysphagia or esophageal dysmotility; Elevated serum creatin kinase (CK), dehydrogenase lactate (DHL), aspartate aminotransferase (ASAT/AST/SGOT) or alanine aminotransferase (ALAT/ALT/SGPT). Which classified her as a definite idiopathic inflammatory myopathy with a ≥90% diagnostic likelihood, and a 7.5 added score without biopsy and was admitted to fulfill diagnostic protocol and start treatment.

The patient had dysphagia as severity criteria, so after ruling out possible infections we initiated pulses of methylprednisolone for 3 days and after that oral prednisone 1mg/kg/day and intramuscular methotrexate 25mg/week.

During her hospital stay we monitored the muscle enzymes and autoantibodies detection by semi automatized immunoblot technique (Tables 1 and 2 respectively), and because of her age group and the known association between dermatomyositis and cancer we decided to run cancer screening by doing seric tumor markers and a torachoabdominal and pelvic tomography that showed no sign of neoplasm.

**Table 1:** Muscle enzymes during patient’s hospitalization

	CPK	AST	ALT	DHL
05.28.2021	3368	172	148	853
06.23.2021	2303	134	138	774
06.24.2021	1196	120	122	670
06.26.2021	1073	76	102	563
07.01.2021	1935	106	113	539
07.02.2021	1848	99	111	509
07.05.2021	1962	114	128	559

**Table 2:** Positive auto-antibodies found

Antibody	Outcome	Intensity
Anti-Mi2 Alpha	Positive	+ +
Anti- Mi2 Beta	Positive	+ +

Electromyography reported changes compatible with active denervation, motor unit potentials with a myopathic pattern and early recruitment with a predominance in proximal muscles in both upper and lower extremities. Motor and sensitive neuroconduction study came out within normal limits (Table 3).

**Table 3:** Electromiography

Spontaneous and/or volitional activity						
Muscle	Activity	Fibers	Wavess +	Fas´c	Poly	Amp
R. Deltoides	Increased	3 +	3 +	None	Many	449
R. Braquial Biceps	Increased	1 +	1 +	None	Few	1514
Right Triceps	Increased	3 +	3 +	None	Many	1037
Right Com. Dig. Extn	Increased	2 +	2 +	None	Many	1487
Right 1st Int Dorsal	Increased	None	None	None	Few	996
Left Vastus Lat	Increased	2 +	1 +	None	None	1042
Left Anterior Tibialis	Increased	2 +	1 +	None	Few	1016
C. Med. Gastroc	Normal	None	None	None	None	637

**Fig. 2:**
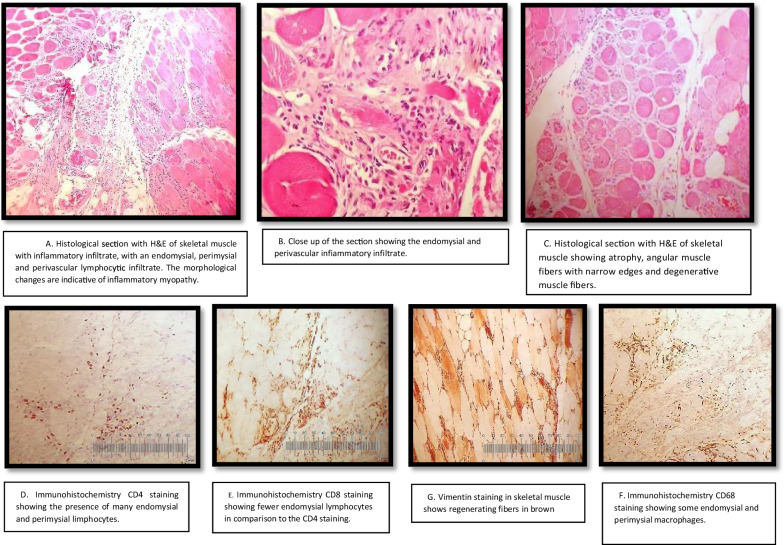
**a** Histological section with H&E of skeletal muscle with inflammatory infiltrate, with an endomysial, perimysial and perivascular lymphocytic infiltrate. The morphological changes are indicative of inflammatory myopathy. **b** Close up of the section showing the endomysial and perivascular inflammatory infiltrate. **c** Histological section with H&E of skeletal muscle showing atrophy, angular muscle fibers with narrow edges and degenerative muscle fibers. **d** Immunohistochemistry CD4 staining showing the presence of many endomysial and perimysial limphocytes. **e** Immunohistochemistry CD8 staining showing fewer endomysial lymphocytes in comparison to the CD4 staining. **g** Vimentin staining in skeletal muscle shows regenerating fibers in brown. **f** Immunohistochemistry CD68 staining showing some endomysial and perimysial macrophages

Left deltoid muscle biopsiy showed characteristic findings with perivascular and endomysial linfocytic infiltrate with regenerative and atrophic muscle fibers without necrosis, compatible with dermatomiositis (Fig. 2a–g).

The patient showed adequate clinical response to the high dose of glucocorticoids treatment. Three days after her admission she tolerated oral intake to both solids and liquids and the MMT8 score increased to 52/75 as well as showing shrinking of the dermatosis and no active lesions. She was discharged 7 days after her admission with prednisone and Methotrexate as immunosuppressive agent. A month later in her follow-up visit we found an improvement in his muscle weakness as noted in her MMT8 and CK levels.

## Discussion and conclusion

The global SARS-CoV2 pandemic has been a challenge to the health authorities and medical science worldwide and it has made us create new protocols to study possible therapies and vaccines.

Is appropriate to remain cautious and vigilant about safety aspects of the COVID-19 vaccines, especially when many of them use new kind of technology (mRNA vaccines) [15]. This fear could be justified by the fact that there has been described more adverse effects in fully vaccinated people who previously had COVID-19 in comparison to those who are just partially vaccinated (1 dose only) and previously had COVID-19. The antibody production between these two groups is very similar which means these patients could receive only one dose to diminish the incidence of adverse effects associated to vaccination [16].

Finally, we agree that there is an association between myopathies and vaccination (at least a temporal one) and there are few case reports about the SARS-CoV2 vaccine, but these cases are extremely rare and the risk is greatly overweighed by the benefits against this new virus, that’s why vaccination should continue to be encouraged [15].

## Data Availability

Authors declare the data to support the findings in this study are available in the article.

## Abbreviations

COVID-19: Coronavirus disease 2019
SARS-CoV 2: Severe acute respiratory disease coronavirus 2
BMI: Body mass index
VAS: Visuale analogue scale
mmHg: Millimetre of mercury
bpm: Beats per minute
MMT8: Manual muscle testing 8
EULAR: European league against rheumatism
ACR: American collegue of rheumatology
CK: Creatin kinase
DHL: Dehydrogenase lactate
ASAT/AST/SGOT: Aspartate aminotransferase
ALAT/ALT/SGPT: Alanine aminotransferase
R. Deltoides: Right deltoids
R. Braquial Biceps: Right Braquial Biceps
Right Com. Dig. Extn: Right common digital extensor
C. Med. Gastroc: Calf medial gastrocnemius
H&E: Hematoxylin and eosin
IIM: Idiopathic Inflammatory myopathies;
DM: Dermatomyositis
PM: Polymyositis
NAM: Necrotizing autoimmune myopathy
IBM: Inclusion bodies myopathy
VAERS: Vaccine Adverse Event Reporting System
USA: United States of America
mRNA: Messenger RNA
